# Nonpainful hyperkeratotic variant of transient lingual papillitis: A case report

**DOI:** 10.1016/j.jdcr.2025.04.008

**Published:** 2025-04-21

**Authors:** Maryum Ali, Shiloh Bass, Robyn Siperstein

**Affiliations:** a Medical Student, Ohio University, Heritage College of Osteopathic Medicine, Athens, Ohio; b College Student, University of Miami, Miami, Florida; c Associate Clinical Professor, Department of Medicine, Florida Atlantic University, Boca Raton, Florida

**Keywords:** fungiform papillae, inflammatory hyperplasia, lingual papillitis, oral inflammatory disease, oral pathology

## Introduction

First described by Whittaker in 1996, transient lingual papillitis (TLP) is an underdiagnosed, inflammatory hyperplasia of fungiform papillae, most commonly on the anterior dorsal aspect of the tongue.^1^^,^^2^ Over the years, subtypes of this condition have been recorded in literature. The classic form presents as painful raised red or white bumps usually towards the tip of the tongue, often abrupt in onset and self-limiting with largely idiopathic etiologies.^2^^,^^3^ However, some episodes have been attributed to stress, hormonal changes, gastrointestinal upset, orthodontic appliances, dental products, smoking, and hypersensitivity to specific food or drinks.^4^

In contrast to the classic variant, the hyperkeratotic or papulokeratotic variant presents as non-painful white bumps or projections on the tongue, due to selective hyperkeratosis seen in inflamed fungiform papillae.^3^ These lesions present abruptly and are also self-limiting, usually in isolated occurrences. However, a subset of patients experience recurrences with repeated exposure to the inciting agent, as seen in this case. In this paper, we describe a case of a recurrent nonpainful hyperkeratotic variant form of TLP, caused by consumption of specific gum flavors. This report provides insight into this underreported condition enabling dermatologists to recognize potential presentations and properly diagnose and educate their patients.

## Case report

**Fig 1:**
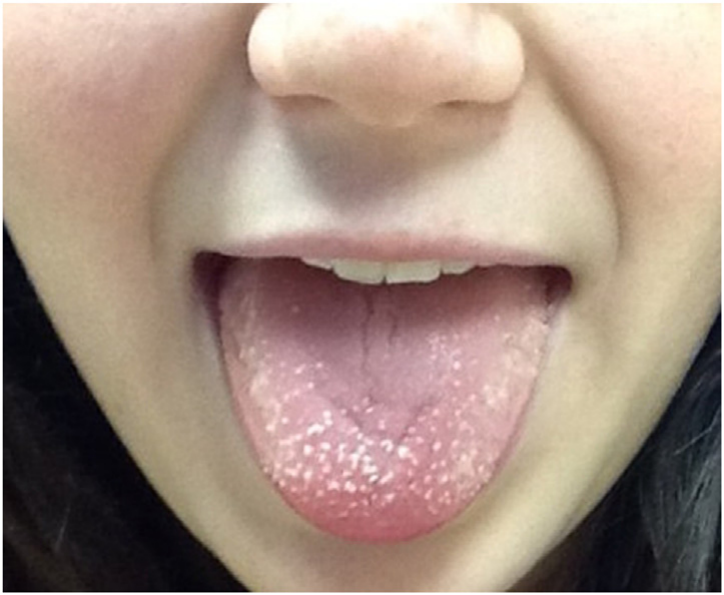
14-year-old with *white*, nonpainful hyperkeratotic *white* papules on the dorsal edge and apex of the tongue diagnosed as hyperkeratotic transient lingual papillitis.

A 14-year-old female presented to her pediatrician with complaints of nonpainful bumps on her tongue. The pediatrician prescribed a compounded solution of diphenhydramine, hydrocortisone, nystatin, and tetracycline (magic mouthwash) which the patient tried for several days without a change in the condition. The bumps persisted for 10 days, and then resolved without further intervention. When she experienced a second eruption, she presented to her dermatologist who recommended a biopsy for diagnosis, however the parent declined. Over the next 6 months, the patient had 6 more episodes, each lasting 1 to 2 weeks. During this time, she stopped using her orthodontic appliances, but still had multiple recurrences of the condition. She subsequently presented to a second dermatology office for another opinion. On exam, there were multiple nonpainful white hyperkeratotic papules located on the dorsal lateral edge and apex of the tongue (Fig 1). There was no fever or lymphadenopathy, and the patient was otherwise healthy, taking no medications. After the case was discussed with multiple colleagues, she was diagnosed with a hyperkeratotic form of TLP. Once the patient was educated on the condition, she was able to narrow down her outbreaks to the use of certain flavored gums like cinnamon, hot mint, and pineapple. Since eliminating those flavored gums, the condition has not recurred.

## Discussion

Hyperkeratotic TLP has yet to be fully understood, as the condition remains underreported due to its transient nature. Since lesions last for only a few days at a time and are often isolated occurrences, most individuals do not present for evaluation.^2^ However, even in cases that do present to the clinic, since most physicians are not well educated on oral conditions, there are many missed diagnoses as occurred to this patient twice. Furthermore, biopsy of these lesions does not always provide unique insight and is not required; however, it often shows inflamed fungiform papillae with associated neutrophilic inflammation of the epithelium.^5^

Variations in this condition, such as color, size, and location of inflammatory papillae, as well as differences in symptoms and time to resolution, make it challenging for medical providers to properly diagnose these patients. This case presents a rare example of the nonpainful hyperkeratotic variant of this condition, as most cases present with painful lesions.^2^ 1 other published report records a non-painful variant of lingual papillitis in a 6-year-old female, although her diagnosis was of eruptive lingual papillitis, which is another form affecting both children and their families. Eruptive lingual papillitis is thought to be due to a viral etiology, causing transient episodes throughout life which can often get mistaken for TLP.^6^

Treatment of these conditions has not yet been fully optimized, as symptom management has been the primary goal.^5^ Most often, mouthwashes, antihistamines, and local anesthetics are used with topical corticosteroids, in addition to consuming cold foods and avoiding the irritants.^5^ All of these management techniques are aimed at suppressing inflammation, rather than targeting the underlying cause.^5^ Avoidance of irritating agents was the main form of management that was recommended to the patient in this case, as her outbreaks were attributed to the gum that she was chewing.

As an underdiagnosed and underreported condition, this case serves as a reminder of the important role of dermatologists in the diagnosis and management of oral pathologies. Current literature states that oral and perioral pathologies are better diagnosed by dermatologists when compared to orthodontists, pediatricians and family doctors.^7^ For this reason, dermatologists should continue to be educated on oral pathologies as the field itself is defined as a branch of medicine concerned with the diagnosis, treatment, and prevention of diseases of the skin, hair, nails, and oral cavity. Our case further highlights the importance of understanding the presentation of TLP and the role of dermatologists in the diagnosis and management of these conditions.

## Conflicts of interest

None disclosed.
